# A Novel Reduced Graphene Oxide Modified Carbon Paste Electrode for Potentiometric Determination of Trihexyphenidyl Hydrochloride in Pharmaceutical and Biological Matrices

**DOI:** 10.3390/s21092955

**Published:** 2021-04-23

**Authors:** Josip Radić, Maša Buljac, Boštjan Genorio, Ema Gričar, Mitja Kolar

**Affiliations:** 1 Department of Environmental Chemistry, Faculty of Chemistry and Technology, R. Boškovića 35, 21000 Split, Croatia; jradic@ktf-split.hr (J.R.); masa@ktf-split.hr (M.B.); 2 Department of Chemical Engineering and Technical Safety, Faculty of Chemistry and Chemical Technology, University of Ljubljana, Večna pot 113, 1000 Ljubljana, Slovenia; bostjan.genorio@fkkt.uni-lj.si; 3 Department of Chemistry and Biochemistry, Faculty of Chemistry and Chemical Technology, University of Ljubljana, Večna pot 113, 1000 Ljubljana, Slovenia; ema.gricar@fkkt.uni-lj.si

**Keywords:** potentiometry, carbon paste electrode, reduced graphene oxide, trihexyphenidyl hydrochloride, pharmaceuticals, urine samples

## Abstract

A novel promising carbon paste electrode with excellent potentiometric properties was prepared for the analysis of trihexyphenidyl hydrochloride (THP), the acetylcholine receptor and an anticholinergic drug in real samples. It contains 10.2% trihexyphenidy-tetraphenylborate ionic pair as the electroactive material, with the addition of 3.9% reduced graphene oxide and 0.3% of anionic additive into the paste, which consists of 45.0% dibutylphthalate as the solvent mediator and 40.6% graphite. Under the optimized experimental conditions, the electrode showed a Nernstian slope of 58.9 ± 0.2 mV/decade with a regression coefficient of 0.9992. It exhibited high selectivity and reproducibility as well as a fast and linear dynamic response range from 4.0 × 10^−7^ to 1.0 × 10^−2^ M. The electrode remained usable for up to 19 days. Analytical applications showed excellent recoveries ranging from 96.8 to 101.7%, LOD was 2.5 × 10^−7^ M. The electrode was successfully used for THP analysis of pharmaceutical and biological samples.

## 1. Introduction

Trihexyphenidyl hydrochloride (THP), the muscarinic acetylcholine receptor antagonist [1], is a synthetic anticholinergic drug that selectively blocks the cholinergic nerve pathway of the striatum [2]. It was approved by the Food and Drug Administration in 2003 for the symptomatic treatment of Parkinson’s disease [3]. It is also commonly used to reduce extrapyramidal side effects occurring during antipsychotic treatment. Typically, patients receive 2–15 mg of the drug daily. It is reported that the drug does not accumulate in tissues [4], as 60% of the dose is rapidly excreted in the urine [5]. Therefore, the expected drug concentration in urine is 2.4 × 10^−6^ to 1.8 × 10^−5^ mol L^−1^.THP is a white powder, soluble in chloroform and methanol, sparingly soluble in methylene chloride and slightly soluble in water [6]. THP is chemically described as 1-cyclohexyl-1-phenyl-3-(1-piperidyl)-1-propanol hydrochloride with molecular formula C_20_H_31_NOxHCl and molar mass of 337.9 g mol^−1^.

The therapeutic importance of THP has led to the development of several analytical methods for its determination and quantification, both in pharmaceutical formulations and biological fluids, including gas chromatography [7,8,9,10], either reversed-phase high performance liquid chromatography [11,12] or high-performance liquid chromatography [13,14], liquid chromatography coupled with mass spectrometry [15,16,17], spectrophotometry [6,18], radioimmunoassay [4], capillary electrophoresis [19,20,21], gravimetry [22], and polarograpy [23]. The use of most of the above techniques usually requires several time-consuming manipulation steps, trained personnel to work on the instruments, and expensive equipment. On the other hand, potentiometric sensors have been widely used for the determination of various species due to their relatively high sensitivity, selectivity, rapid response, applicability to turbid and colored solutions, adaptability to on-line analytical systems, and small sample volume [24,25,26,27,28,29,30]. Therefore, ion-selective electrodes have found applications in clinical, industrial, and environmental analysis [31]. One of the most common procedures for the development of ISE (for the determination of drugs, whether the active ingredient is a component of pharmaceutical preparations or biological fluids) is the incorporation of a lipophilic ion-pair-associated complex into a polyvinyl chloride (PVC) based membrane [31,32,33,34,35] or the preparation of carbon paste electrodes (CPE) [5,24,30,31,36,37,38,39,40,41,42,43,44].

However, PVC based membranes exhibit several disadvantages, such as leaching of plasticizers and membrane ionophores, limited adhesion of supporting materials in addition to high water uptake [45,46]. On the other hand, CPE are easier to fabricate. They show faster responses with simple surface renewability and also have lower ohmic resistance [41].

In the literature, there is only one work on the determination of THP using a carbon paste electrode [5]. Since nanomaterials are playing an increasingly important role in the fabrication of chemo- and bio-sensors [24,31,47,48,49,50] due to the possibility of their direct and easy incorporation into ion-selective membranes, synthesized reduced graphene oxide (rGO) was used in this work. A sensor for the determination of THP containing any type of nanomaterials has not yet been developed according to the available literature. rGO is a widely used component in electrochemical measurements due to its uncomplicated synthesis, high electrical conductivity, light weight, high specific surface area, strong mechanical strength, and chemical stability [24,28,51,52,53]. Additionally, when compared to graphene it is significantly cheaper, while it exhibits similar properties, and it is unquestionably more conductive than graphene oxide and as such the most appropriate choice to use in electrochemical sensing applications.

The present study describes the development and application of a carbon paste electrode chemically modified with trihexyphenidyl for the selective and sensitive determination of THP in pure solutions, pharmaceutical (Parkopan tablets) and biological (urine) samples. The results confirm that the prepared electrode exhibits excellent selectivity for THP, wide concentration range, low detection limit, fast response time and reproducibility, which are the most important performance factors of the electrode besides the absence of special pretreatment steps.

## 2. Materials and Methods

### 2.1. Equipment

Morphology characterization of graphite oxide (GO) and rGO was performed using a scanning field emission electron microscope Zeiss ULTRA plus FE-SEM (Jena, Germany). SEM images were acquired at 2 kV using InLens or SE2 detector at WD 5.5 mm. Elemental analysis of the samples was done inside SEM using EDS analysis with an Oxford X-Max SDD detector (Wycombe, UK), processed with INCA software (Wycombe, UK). EDS analysis was carried out at 20 kV.

Specific surface area (m^2^/g) was measured by N_2_ adsorption at 77 K on an ASAP 2020 Micromeritics (Norcross, GA, USA) instrument using the BET analysis method. Samples were degassed under vacuum (5 millitorr) at 120 °C for 2 h.

Microanalyses were performed by combustion analysis on a Perkin-Elmer CHN Analyzer 2400 II.

Potentiometric measurements were performed using the digital pH/mV meter model Seven Easy (Mettler-Toledo, GmbH, Schwerzenbach, Switzerland). The electrochemical cell was completed with an external Ag/AgCl reference electrode, model InLab^®^ Reference—51343190 (Mettler-Toledo GmbH, Schwerzenbach, Switzerland). A Premium hotplate stirrer model MSH-20A (Witeg Labortechnik, GmbH, Wertheim, Germany) was used for stirring the solutions.

Measurements of the pH values of the solutions were performed using a pH electrode InLab Expert Pro (Mettler Toledo GmbH, Greifensee, Switzerland). 

### 2.2. Chemicals and Materials

All chemicals and reagents were of the analytical grade and double distilled deionized water was used throughout experiments. 

Graphite flakes (Timrex KS 44) were purchased from Imerys Graphite & Carbon (Bodio, Switzerland). Dibutylphthalate (DBP), phosphotungstic acid hydrate (PTA), maprotiline hydrochloride, and THP were purchased from Sigma (USA). Sodium tetraphenylborate (NaTPB), zinc nitrate hexahydrate, magnesium nitrate hexahydrate, acetylsalicylic acid, glucose, galactose, and fructose were purchased from Merck (Germany). Paracetamol was kindly provided by Galenic laboratory Split—Dalmatia County Pharmcy (Croatia). Sodium acetate anhydrous was purchased from Gram-mol (Croatia); glacial acetic acid, potassium nitrate, calcium nitrate tetrahydrate, iron (III) nitrate nonahydrate, and lead(II) nitrate from Kemika (Croatia); silver nitrate and ammonium chloride from Sigma-Aldrich (Germany). Parkopan (2 mg/tablet) used in the application of the prepared electrode was obtained from the local drugstore.

### 2.3. Procedures

Stock solutions of 1.0 × 10^−2^ M THP were prepared in a 50 mL volumetric flask by dissolving 168.9 mg of THP a small volume of acetate buffer heated to 30 °C and making up the volume with acetate buffer at room temperature. Working solutions were prepared by serial dilution. Interfering stock solutions of 1.0 × 10^−2^ M for each of K^+^, NH_4_^+^, Ca^2+^, Mg^2+^, Zn^2+^, Pb^2+^, Fe^3+^, glucose, galactose, fructose, acetylsalicylic acid, paracetamol, and maprotiline hydrochloride were prepared by dissolving the appropriate amount of the compounds and subsequent dilutions, where necessary. All solutions and dilutions were prepared in a 1.5 × 10^−2^ M solution of acetate buffer pH 4 by diluting glacial acetic acid (0.570 mL) and sodium acetate (452 mg) in distilled water using a 1000 mL volumetric flask.

**Figure 1:**
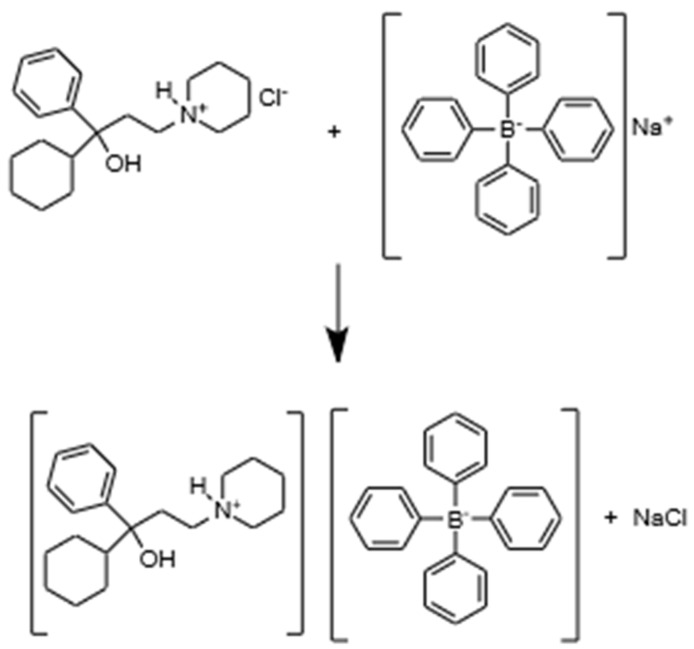
Proposed schematic diagram of reaction between THP × HCl and NaTPB ion pair associated complex as sensing element.

THP has a high affinity for forming IAC with the oppositely charged ion pairing agent NaTPB (Figure 1). The precipitated ion pair associated complex (IAC) was used as the sensing material of the electrode.

It was prepared by dissolving 67.6 mg of THP in 20 mL of distilled water heated to 30 °C. A solution of NaTPB was prepared by dissolving 68.5 mg of NaTPB in 20 mL of distilled water. Twenty mL of the THP solution was slowly added to 20 mL of the NaTPB solution. The resulting white precipitate was filtered, washed thoroughly with distilled water (until negative reaction to chloride ions), covered, and dried at room temperature for at least 72 h. The dried precipitate was then ground to obtain a fine powder which was used as a sensing element in the electrode.

The chemical compositions of the precipitate were confirmed by elemental CHN analysis. The C, H, and N contents of the trihexaphenidyl-tetraphenylborate ion associated complex (THP-TPB) were 85.14%, 7.85%, and 2.20%, respectively. Compared to the corresponding calculated values of 84.99%, 8.45%, and 2.25%, this confirms that the combination rate of the prepared THP-TPB ion pair was 1:1.

GO was synthesized by the improved Hummer’s method [54]. KMnO_4_ (10 g) was slowly added to a mixture of H_2_SO_4_ (357 mL) and H_3_PO_4_ (40.2 mL). Upon cooling to room temperature, 10 g of graphite flakes was added to the reaction mixture. Over the next five days, KMnO_4_ was added in 6 aliquots. The reaction was then quenched by addition of 500 mL of crushed ice and H_2_O_2_ (30 mL). The mixture was transferred to plastic centrifuge bottles, diluted with H_2_O, and centrifuged (4100 rpm for 30 min). The supernatant was decanted and the remaining solid washed with H_2_O and centrifuged (4100 rpm for 30 min). This washing–centrifuging cycle was performed until the supernatant reached the pH value of 5.0 or more. The remaining material then was freeze-dried and stored at room temperature.

Dried GO was dissolved in purified water using an ultrasonic bath and placed under a water-cooled reflux system with continuous stirring. Twenty mL of hydrazine hydrate was slowly added to carry out the reduction of GO. The mixture was heated in an oil bath at 100 °C for 24 h. rGO, a black solid, was then filtered hot and washed with 500 mL boiling water. The synthesized reduced graphene oxide (rGO) was further used for the fabrication of electrodes. 

The electrodes were investigated by varying the ratios (*w*/*w*) of graphite, IAC, rGO, solvent mediator, and anionic additive, until the optimum composition that exhibits the best response characteristics was achieved. According to the literature, all the components were made homogenous in an agate mortar [36,45]. The obtained smooth paste was carefully packed into a laboratory made polytetrafluoroethylene electrode body (Teflon holder) (Figure S1, Supplementary Materials). Before use, the external surface of the carbon paste was polished with circular motions on parafilm until the surface had a shiny appearance. Finally, the electrodes were conditioned by soaking in an acetate buffer solution for 1 h before potentiometric measurements. 

All potentiometric measurements by the MCPE were performed with the following cell configuration at room temperature:

Ag/AgCl (saturated)//sample solution/carbon paste/stainless-steel rod.

For calibration, the conditioned electrodes were separately immersed in 20 mL of each of the working standard solutions. The properties of the electrodes were investigated by measuring the change in the potential value of the THP solutions whose concentrations were changed in an ascending manner from the lowest of 6.3 × 10^−8^ to the highest one of 1.0 × 10^−2^ M. Potential readings were plotted as a function of the negative logarithm of the THP concentration. An electrode that showed the Nernstian slope and the widest linear range was used for further examination of the electrode properties. 

THP was determined in 50 mL of the prepared aliquot by the standard addition method. Data were plotted as the numerical expression $10^{\frac{E}{S}}\times \left(V_{A}+V_{s}\right)$ versus $\frac{c_{s}\times V_{s}}{V_{A}}$, where $V_{A}$ was the concentration of the tested aliquot, and $V_{s}$ was the sum of increments of the standard THP solution added in the aliquot for each recorded potential change. The concentration of the standard THP solution added in the aliquot, the slope of the calibration curve, and the recorded potential were labeled as $c_{s},\;S,$ and E, respectively. 

The intersection of the extrapolated segment of the obtained graph and $\frac{c_{s}\times V_{s}}{V_{A}}$-axis represented the negative value of the concentration of the THP in the aliquot.

The fabricated electrode was immersed in a 50 mL aliquot and the potential was recorded. A volume of 50 mL aliquot with concentration of 1.0 × 10^−4^ M and 1.0 × 10^−5^ M was titrated against 3.3 × 10^−5^ M solution of PTA and 1.0 × 10^−3^ M solution of NaTPB, respectively, with small increments from 25 µL to 250 µL of titrant. The potentials were monitored after each addition and used to plot the titration curve and consequently calculate the THP concentration. 

Twenty-five weighed Parkopan tablets (50.0 mg of THP) were pulverized in a mortar and thoroughly transferred to 500 mL of acetate buffer solution previously heated to 30 °C. The mixture was shaken for about 60 min and filtered. The resulting filtrate (3.3 × 10^−4^ M of THP) was diluted to obtain 1.0 × 10^−4^, 1.0 × 10^−5^ and 1.0 × 10^−6^ M of THP, respectively.

Twenty-five mL urine samples were spiked with 50 µL, 500 µL, and 5 mL of 1.0 × 10^−3^ M THP, respectively, and transferred to 50 mL volumetric flasks. The volumes were made up to the marks with acetate buffer to obtain 1.0 × 10^−4^, 1.0 × 10^−5^ and 1.0 × 10^−6^ M of THP, respectively.

## 3. Results and Discussion

### 3.1. Characterization of GO and rGO

**Figure 2:**
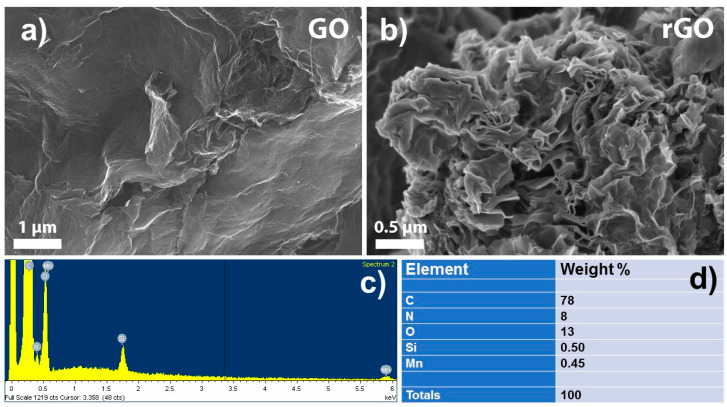
SEM images of: (**a**) graphite oxide (GO) and (**b**) reduced graphene oxide (rGO). EDS analysis of reduced graphene oxide (rGO): (**c**) EDS spectra and (**d**) quantification.

GO and rGO were morphologically characterized by SEM. Figure 2a,b clearly shows the layered structure of the GO and rGO with high specific surface area. The flakes have a diameter of several µm and are highly wrinkled, especially rGO. Moreover, the rGO flakes have porosity inside the worm-shaped particles, which is advantageous for sensing applications. The existence of worm-shaped particles was confirmed with XRD analysis (Figure S2). The high specific surface area of rGO was confirmed by BET analysis and was 369 m^2^/g. However, the specific surface area of GO was 10 times smaller (37 m^2^/g) than that of rGO, indicating that chemical reduction of GO provides additional exfoliation of rGO. EDS analysis of rGO revealed the chemical composition of the material (Figure 2c), quantification (in Figure 2d), and that the majority of oxygen functionalities were reduced during the reduction of GO. The latter indirectly suggests that the graphene structure was partially restored. Thorough elemental analysis was performed using ICP-MS (Table S1). Interestingly, we identified residual nitrogen (8 wt%) that could be incorporated during the reduction with hydrazine and residual Mn impurities (<1 wt%) introduced during the oxidation step with KMnO_4_. Both elements were reported to be beneficial for enhanced sensing applications as discussed in previous reports [55,56,57]. Additionally, Raman (Figure S3), TGA (Figure S4), FTIR (Figure S5), and XPS (Figure S6 and Table S2) experiments were performed to further characterize the prepared materials.

### 3.2. Potentiometric Measurements

#### 3.2.1. Optimization of Carbon Paste Electrode Composition

In general, and from reported articles, it is evident that both the selected organic compound [38,39,58,59], usually used as a precipitating agent for IAC, and binder [30,37,39,40] strongly influence the response of CPE and its characteristics. Various studies and citations can be found in the literature with binder proportions, but all of them in a range between 15 and 55 wt% [27,30,36,58,60]. In this work, NaTPB was used as the anionic precipitating agent, since it was reported that the most suitable CPE electrodes for the determination of THP are those that contain the THP-TPB as IAC [5] in their composition. Therefore, DBP was chosen as the solvent mediator. However, according to the available literature, reduced graphene oxide has not yet been used in CPEs for the determination of THP.

The electrode characteristics were measured using different ratios of carbon paste components. The response characteristics of different CPEs to obtain the lowest possible limit of detection (LOD), wider linear range, and higher regression coefficient are illustrated in Table 1. The unmodified CPE displayed no measurable response to THP (no. 1–4). Electrodes no. 6–13 were prepared using THP-TPB as the recognition ion-pair. Variable amounts of IAC were tested to find the best composition. The results clearly showed that there are no significant changes in the response characteristics of the electrodes containing between 1 and 14.5 wt% of IAC. However, electrodes with 8.1 and 10.2 wt% of IAC showed a slightly higher slope. 

**Figure 3:**
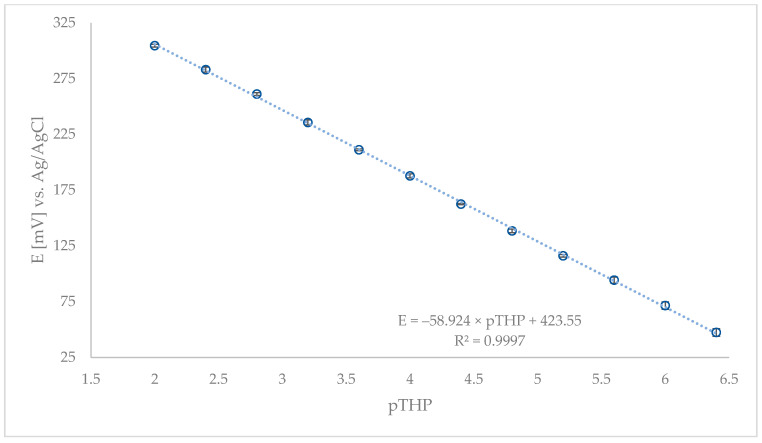
Calibration graph of the optimized—no. 24 CPE.

Based on the published results [5,30,39,40,61], a sufficient amount of lipophilic additive was added in this study to further improve the CPE characteristics (no. 14–20.) The presence of 0.1 or 0.3 wt% of NaTPB, increased the sensitivity and linear range of the electrode. A change in the slope value from −50.2 to −54.3 or −54.6 mV/decade and from −50.0 to −55.1 or −55.7 mV/decade was observed, respectively. CPE saturated with NaTPB as a lipophilic additive (no. 16, 17 and 20) showed a lower slope. Finally, the addition of synthesized rGO significantly improved the electrode characteristics and increased the sensitivity to a Nernstian slope of −58.9 mV/decade (no. 24, Figure 3). As a result, 40.6% graphite, 45.0% DBP, 10.2% IAC, 0.3% NaTPB, and 3.9% rGO were used as the best composition to prepare CPE for further measurements.

#### 3.2.2. The Influence of pH on the Electrode Response

**Figure 4:**
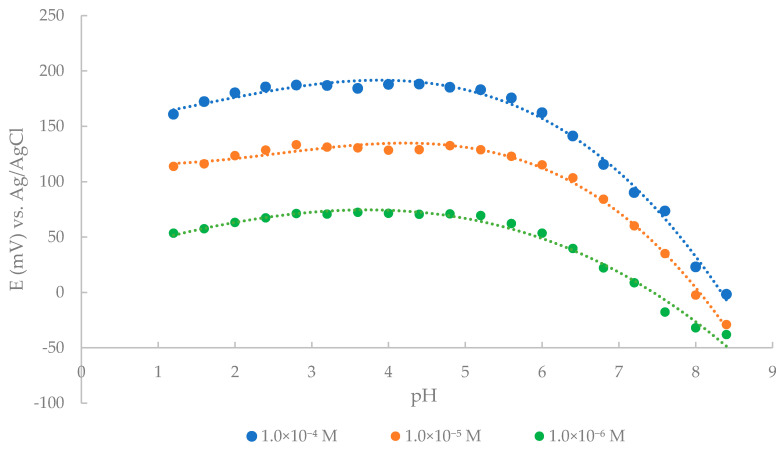
Effect of pH on the potential response of the proposed CPE.

pH changes on the CP electrode response were carried out in 1.0 × 10^−4^, 1.0 × 10^−5^ M, and 1.0 × 10^−6^ M THP solutions, varying the pH from 1.2 to 8.4 by small additions of HCl or NaOH solutions. The result obtained shows that the electrode can be safely used for THP determination over the pH range of 2.4–5.2, as shown in Figure 4. As can be seen, the potential became gradually lower above pH 5.2, which may be caused by the OH^−^ ions penetrating the membrane and forming THP free base and consequently decreasing the amount of protonated species in the test solutions. One of the explanations of why the potential also decreases at pH values below 2.4, could be that H_3_O^+^ ions can compete with THP ions due to their high mobility and thus interfere with the measuring signal [5]. No significant deviations of the described pH effect were observed at different concentrations of THP.

#### 3.2.3. Dynamic Response Time, Reversibility, and Lifetime of the Electrode 

The results on the determination of the response time, reversibility, and hysteresis of the electrodes are also of great importance for the subsequent analytical applications. The practical dynamic response time and reversibility of the prepared electrode were tested by measuring the time required to reach a steady-state potential value within ±1 mV after successive immersions of the electrode in a series of THP solutions, each of which had a 10-fold increase in concentration from 1 × 10^−6^ to 1 × 10^−2^ M. As can be seen, the electrode reached a steady potential in a particularly short time (within ≤5 s). 

**Figure 5:**
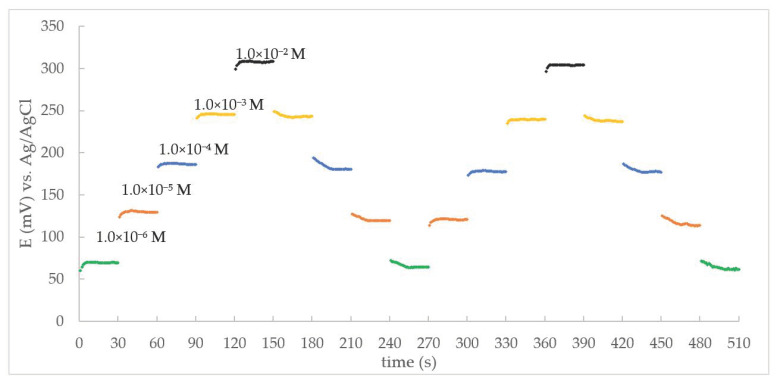
Dynamic response time and reversibility of the proposed CPE.

To assess reversibility, the same procedure was performed but in the reverse direction (from high to low THP concentration). The results showed that the electrode required a longer time (within ≤13 s) to respond to changes in analyte concentration and to reach equilibrium values assuming residual THP was still adsorbed on the electrode surface. The electrode was reversible and showed no memory effect toward previous solutions with higher THP concentration (Figure 5). After four measurement cycles, the electrode became unusable while the electrode sensitivity was lost due to permeability and disintegration of the surface. This was resolved by renewing the membrane surface as previously described.

The lifetime of the proposed CPE was investigated by periodically calibrating it in THP solutions and calculating the slope. Figure S7 in the supplementary materials shows that no remarkable change was observed in the slopes of the calibration curves within 19 days. The stability of the proposed CPE is due to the renewability of the surface. Over the next 11 days, as can be seen, there was a decrease in the slope from 57.4 to 49.2 mV/decade.

#### 3.2.4. Interference Studies

Since the samples may contain varying numbers of interfering species, selectivity was investigated as an important factor of the electrode applicability. Interfering ions obstruct the analyte ions and give erroneous results during measurements. Generally, selectivity is the ability of a sensor to discriminate between analyte ions and interfering ions. Selectivity coefficients express the numerical value of selectivity for each interfering species tested separately. They are defined using various equations depending on the chosen IUP AC method. A lower value of the selectivity coefficient means that the electrode can determine the target species without noticeable interference. The selectivity coefficients of the proposed CPE were investigated using the matched potential method (MPM) and estimated using the following equation:

(1) $$K_{\mathrm{THP},\;\mathrm{INT}}^{pot}=\frac{a_{\mathrm{THP}}^{\;\;\;\;\prime }-a_{\mathrm{THP}}}{a_{\mathrm{INT}}}$$

where $a_{\mathrm{THP}}^{\;\;\;\;\prime }$ is the known activity of the added THP solution into a reference THP solution that contains a fixed activity $\left(a_{\mathrm{THP}}\right)$ and $a_{\mathrm{INT}}$ is the activity of the added tested interfering species producing the same potential change.

According to the literature, several compounds were included in the interference study because they are either a common excipient of the medications or an ion present in urine [36,41,59]. Interferences to some nonsteroidal anti-inflammatory analgesics as well as to an antidepressant were also tested. As shown in Table 2, the proposed electrode demonstrated high selectivity toward THP over the tested interfering ions and is accordingly usable for analytical measurements.

#### 3.2.5. Analytical Application and Real Sample Analysis

The accuracy and precision were tested by seven determinations at three different concentration levels in pure solutions. The proposed CPE was applied to determine THP in pharmaceutical preparations and spiked urine samples using the calibration curve method, the standard addition method, and potentiometric titration.

The direct potentiometric determination of THP using the calibration curve gave average recovery of 98.7 ± 1.0%, 97.6 ± 1.7%, and 97.5 ± 1.9% in pure solutions, tablets, and urine, respectively. Despite the fact that this method exhibits higher deviations compared to the other two, the obtained results show a very high accuracy, especially because results of studies obtained at very low THP concentrations in real samples were also included in the statistical analysis.

The standard addition method was used to minimize the potential matrix effect that may have interfered with the analyte, THP, to obtain better recoveries and improved standard deviations of the tested samples. The average recoveries were 99.3 ± 0.4%, 98.7 ± 0.9%, and 101.3 ± 1.0% in pure solutions, tablets, and urine, respectively. The model obtained for this method is shown in Figure S8.

**Figure 6:**
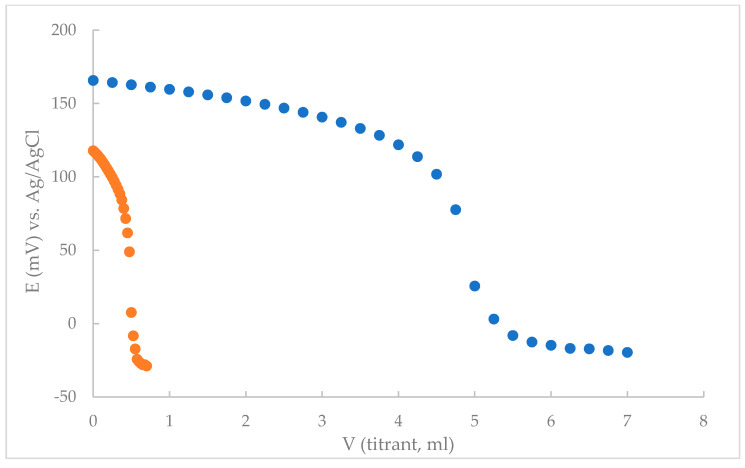
A typical potentiometric titration curve for 50 mL aliquot of Parkopan tablets.

Furthermore, the developed electrode was investigated in conjunction with an Ag/AgCl reference electrode as an end point indicator electrode for titrations of THP with NaTPB or PTA as titrant. The end point of the titration was determined as the mean of the minimum of the first derivatives of seven replicates of the titration curves. The well-defined S-shape of the titration curve and the large potential change at the end point suggest that highly accurate results could be obtained (Figure 6 and Figure S9). The results obtained by this method were similar to those obtained by the standard addition method.

The results of the real sample analysis are summarized in Table 3. The content of THP showed good agreement with the declared (tablets) or expected (spiked urine samples) amounts in the real samples tested as well as in the pure solutions.

## 4. Conclusions

In this work, a novel type of rGO based carbon paste electrode was presented as a useful analytical tool for the rapid, low-cost, and simple determination of THP in pure form and in real samples using the calibration curve method, the standard addition method, and potentiometric titration. SEM and BET were used to characterize the synthesized GO and rGO, respectively. The proposed CPE showed good performance characteristics with minimal sample pretreatment and gave precise and accurate results down to a concentration level of 4.0 × 10^−7^ M. It showed excellent selectivity, Nernstian behavior, reproducibility, stability with fast dynamic response time, and adequate lifetime. The surface of the electrode was easily regenerated. Recommendation to use the described CPE for THP determination even in routine laboratories can be made.

## Figures and Tables

**Table 1:** Carbon paste composition optimization for direct potentiometric determination of THP.

CPE (no.)	Ingredient (%)					Linear Range (mol L^−1^)	Detection Limit (mol L^−1^)	Slope (mV/dec) ± SD *	*R* ^2^
	G	DBP	IAC	NaTPB	rGO				
1	59.8	40.2	-	-	-	-	-	−12.4 ± 0.7	0.8849
2	55.3	44.7	-	-	-	-	-	−17.0 ± 0.9	0.9046
3	51.8	48.2	-	-	-	-	-	−14.6 ± 0.8	0.8963
4	45.2	54.8	-	-	-	-	-	−9.7 ± 0.7	0.8395
5	54.1	45.6	0.3	-	-	6.3 × 10^−6^–1.0 × 10^−2^	4.7 × 10^−6^	−38.1 ± 0.6	0.9892
6	53.5	45.1	1.0	-	-	2.5 × 10^−6^–1.0 × 10^−2^	2.4 × 10^−6^	−45.5 ± 0.3	0.9972
7	52.0	46.5	1.5	-	-	2.5 × 10^−6^–1.0 × 10^−2^	2.0 × 10^−6^	−46.5 ± 0.5	0.9935
8	50.6	46.5	2.9	-	-	2.5 × 10^−6^–1.0 × 10^−2^	2.0 × 10^−6^	−47.8 ± 0.3	0.9976
9	49.8	44.5	5.7	-	-	2.5 × 10^−6^–1.0 × 10^−2^	2.2 × 10^−6^	−48.3 ± 0.6	0.9921
10	47.6	44.3	8.1	-	-	2.5 × 10^−6^–1.0 × 10^−2^	2.2 × 10^−6^	−50.2 ± 0.3	0.9977
11	44.9	44.9	10.2	-	-	2.5 × 10^−6^–1.0 × 10^−2^	2.1 × 10^−6^	−50.0 ± 0.3	0.9980
12	43.3	44.7	12.0	-	-	2.5 × 10^−6^–1.0 × 10^−2^	2.0 × 10^−6^	−49.4 ± 0.3	0.9981
13	40.5	45.0	14.5	-	-	2.5 × 10^−6^–1.0 × 10^−2^	2.4 × 10^−6^	−48.1 ± 0.3	0.9982
14	46.8	45.0	8.1	0.1	-	1.0 × 10^−6^–1.0 × 10^−2^	9.3 × 10^−7^	−53.9 ± 0.2	0.9990
15	46.3	45.4	8.0	0.3	-	1.0 × 10^−6^–1.0 × 10^−2^	7.9 × 10^−7^	−54.5 ± 0.2	0.9993
16	45.4	45.5	8.2	0.9	-	2.5 × 10^−6^–1.0 × 10^−2^	2.3 × 10^−6^	−50.2 ± 0.4	0.9964
17	44.6	44.9	8.3	2.2	-	6.3 × 10^−6^–1.0 × 10^−2^	4.5 × 10^−6^	−46.8 ± 0.5	0.9957
18	44.8	45.0	10.1	0.1	-	1.0 × 10^−6^–1.0 × 10^−2^	6.6 × 10^−7^	−54.6 ± 0.4	0.9970
19	44.1	45.4	10.2	0.3	-	1.0 × 10^−6^–1.0 × 10^−2^	7.1 × 10^−7^	−55.6 ± 0.3	0.9983
20	43.5	45.4	10.1	1.0	-	2.5 × 10^−6^–1.0 × 10^−2^	1.7 × 10^−6^	−51.2 ± 0.4	0.9976
21	44.3	45.3	10.0	0.3	0.1	1.0 × 10^−6^–1.0 × 10^−2^	7.6 × 10^−7^	−55.8 ± 0.4	0.9968
22	43.8	45.4	10.1	0.2	0.5	1.0 × 10^−6^–1.0 × 10^−2^	7.5 × 10^−7^	−56.8 ± 0.2	0.9992
23	42.3	45.2	10.2	0.3	2.0	4.0 × 10^−7^–1.0 × 10^−2^	3.3 × 10^−7^	−58.4 ± 0.2	0.9991
24 ^s^	40.6	45.0	10.2	0.3	3.9	4.0 × 10^−7^–1.0 × 10^−2^	2.5 × 10^−7^	−58.9 ± 0.2	0.9992
25	38.1	45.6	9.9	0.2	6.2	4.0 × 10^−7^–1.0 × 10^−2^	3.6 × 10^−7^	−57.4 ± 0.3	0.9986
26	42.7	44.9	8.1	0.3	4.0	4.0 × 10^−7^–1.0 × 10^−2^	3.7 × 10^−7^	−57.1 ± 0.4	0.9977

* Standard deviation (5 replicates); ^s^—selected carbon paste electrode.

**Table 2:** Selectivity coefficients ($-logK_{\mathrm{THP},\;\mathrm{INT}}^{pot})$ of various ions for proposed CPE.

Foreign Ions	$\left(-logK_{THP,\;INT}^{pot}\right)$
K^+^	−4.19
NH_4_^+^	−4.36
Ca^2+^	−3.81
Mg^2+^	−3.62
Zn^2+^	−3.72
Pb^2+^	−3.18
Fe^3+^	−3.26
glucose	−3.46
galactose	−3.53
fructose	−3.94
acetylsalicylic acid	−4.11
paracetamol	−3.95
maprotiline hydrochloride	−2.84

**Table 3:** Analysis of THP in pure solution, pharmaceutical formulation, and spiked urine sample using different methods.

		Taken THP (mol L^−1^)	Recovery ± RSD * (%)
Pure solutions	Direct method	1.0 × 10^−4^	99.0 ± 0.6
		1.0 × 10^−5^	98.4 ± 1.3
	Standard addition method	1.0 × 10^−4^	99.4 ± 0.4
		1.0 × 10^−5^	99.2 ± 0.5
	Potentiometric titration	1.0 × 10^−4^	99.4 ± 0.3
		1.0 × 10^−5^	99.2 ± 0.6
Parkopan tablets	Direct method	1.0 × 10^−4^	98.1 ± 1.4
		1.0 × 10^−5^	97.0 ± 2.0
	Standard addition method	1.0 × 10^−4^	98.9 ± 0.6
		1.0 × 10^−5^	98.4 ± 1.0
	Potentiometric titration	1.0 × 10^−4^	98.9 ± 0.7
		1.0 × 10^−5^	98.7 ± 1.0
Spiked urine samples	Direct method	1.0 × 10^−4^	98.2 ± 1.7
		1.0 × 10^−5^	96.8 ± 1.8
	Standard addition method	1.0 × 10^−4^	100.9 ± 0.8
		1.0 × 10^−5^	101.7 ± 1.1
	Potentiometric titration	1.0 × 10^−4^	101.0 ± 0.9
		1.0 × 10^−5^	98.6 ± 1.0

* Seven replicates.

## Supplementary Materials

Supplementary Materials are available online at https://www.mdpi.com/article/10.3390/s21092955/s1.
