# Supplementary material for: A Novel Reduced Graphene Oxide Modified Carbon Paste Electrode for Potentiometric Determination of Trihexyphenidyl Hydrochloride in Pharmaceutical and Biological Matrices
Source: Sensors (Basel). 2021 Apr 23;21(9):2955. doi: 10.3390/s21092955 (PMC8122888; doi:10.3390/s21092955)
Supplement: Supplementary file 1 [file sensors-21-02955-s001.zip › sensors-1170104-supplementary.pdf]

Supplementary Materials:

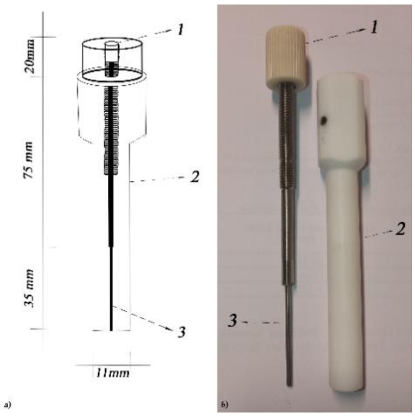

(**a**) (**b**)

Figure 1. (**a**) Scheme of assembled Teflon holder; (**b**) Image of dissembled laboratory made Teflon holder (1-coaxial cable slot, 2-Teflon body, 3-stainless-steel rod of diameter of 2 mm).

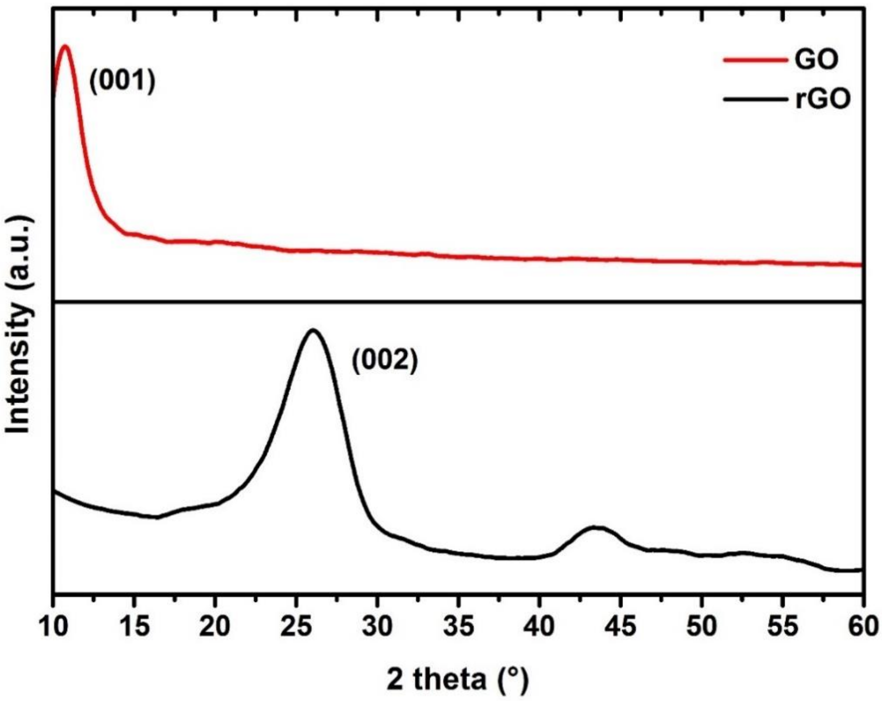

Figure 2. XRD patterns of graphite oxide, (GO)—red curve and reduced graphene oxide, (rGO)—black curve.

Both XRD patterns are typical for GO and rGO materials. The red curve shows only diffraction on (001) crystal planes due to oxygen functionalities and distorted stacking. On the other hand, reduction of GO shows diffraction patterns where (002) and other crystal planes are visible. Such diffraction is typical for worm-like structures.

Table 1. N at% comparison between XPS and C, H, N analysis.

9 Be [He]	11 B [He]	24 Mg [He]	39 K [He]	44 Ca [He]	47 Ti [He]	51 V [He]

W [µg/g]	W [µg/g]	W [µg/g]	W [µg/g]	W [µg/g]	W [µg/g]	W [µg/g]
200.5	25205.1	210.4	4755.4	23.4	6.3	7.7
**52 Cr [He]**	**55 Mn [He]**	**56 Fe [He]**	**59 Co [He]**	**60 Ni [He]**	**63 Cu [He]**	**66 Zn [He]**
W [µg/g]	W [µg/g]	W [µg/g]	W [µg/g]	W [µg/g]	W [µg/g]	W [µg/g]
83.0	404.6	741.2	7.9	12.3	286.0	165.8
**69 Ga [He]**	**75 As [He]**	**85 Rb [He]**	**88 Sr [He]**	**90 Zr [He]**	**95 Mo [He]**	**107 Ag [He]**
W [µg/g]	W [µg/g]	W [µg/g]	W [µg/g]	W [µg/g]	W [µg/g]	W [µg/g]
16.6	117.9	8.0	13.3	31.2	316.3	424.5
**111 Cd [He]**	**137 Ba [He]**	**195 Pt [He]**	**205 Tl [He]**	**208 Pb [He]**	**209 Bi [He]**	**238 U [He]**
W [µg/g]	W [µg/g]	W [µg/g]	W [µg/g]	W [µg/g]	W [µg/g]	W [µg/g]
3.9	10.2	8.5	24.7	10.8	5.8	4.8

Trace metal analysis was performed using the ICP-MS method. The results clearly indicate high concentrations of B, K, Mn, and Fe. Most of the high concentrations of the metals were introduced with the synthesis method, however some of them were inherent to the starting graphite materials (B, Fe, etc.).

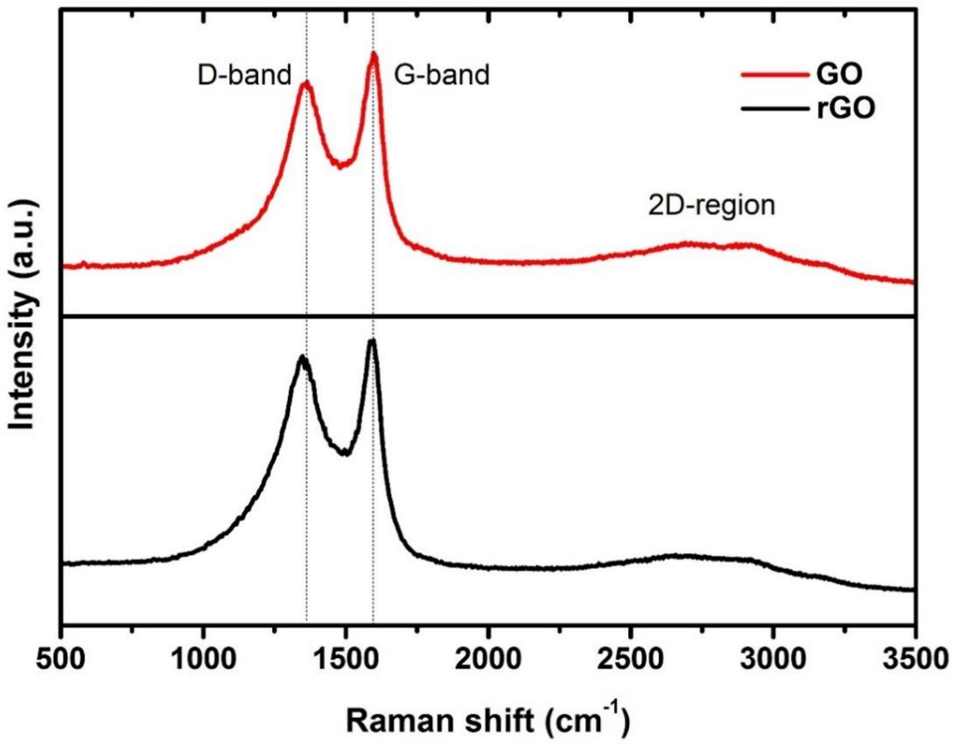

Figure 3. Raman spectra of graphite oxide (GO)—red curve, and reduced graphene oxide (rGO)—black curve.

Both Raman spectra show intense D-band and G-band. High ID/IG ratio indicates highly defected structures in terms of structural and chemical defects.

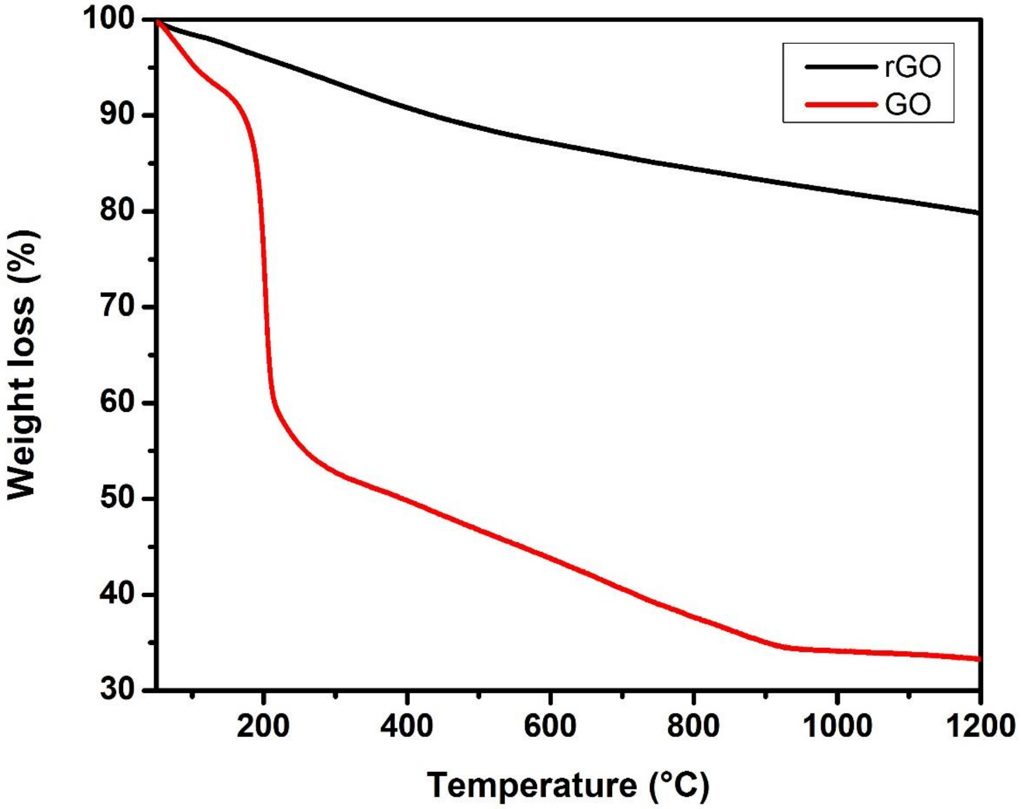

Figure 4. TGA curves of graphite oxide (GO)—red curve, and reduced graphene oxide (rGO)—black curve.

TGA results clearly indicate that the GO that we used as a precursor for preparing rGO is highly oxidized. rGO is however, much less oxidized, which indirectly indicates restoration of sp2-hybridization of the lattice carbon atoms. Also, the big difference between GO and rGO in weight loss, indirectly indicates that the C/O atomic ratio increases upon reduction.

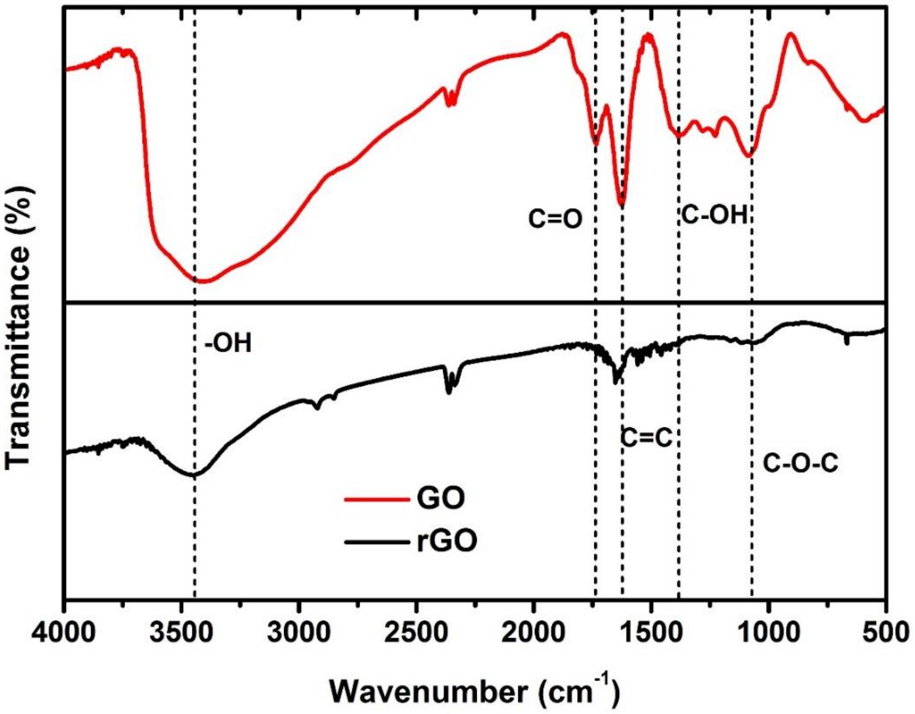

Figure 5. FTIR spectra of graphite oxide (GO)—red curve, and reduced graphene oxide (rGO)—black curve.

The FTIR spectrum of GO (red curve) shows high concentration of oxygen functionalities while the spectrum of rGO (black curve) indicates that during the reduction process the majority of the oxygen functionalities are removed.

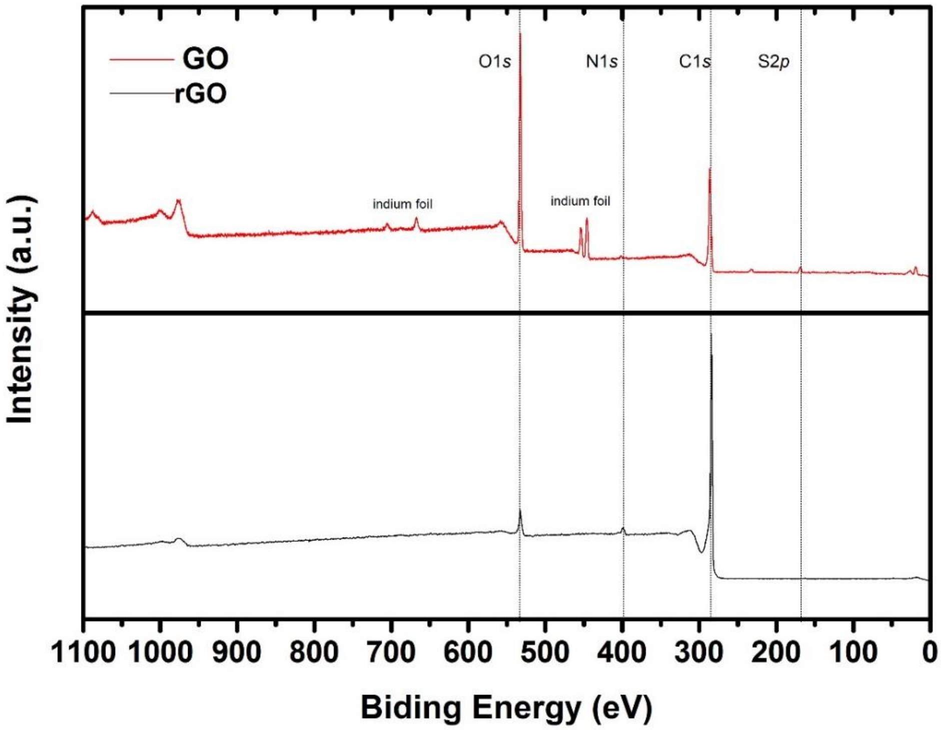

Figure 6. XPS spectra of graphite oxide (GO)—red curve, and reduced graphene oxide (rGO)—black curve.

Table 2. Quantification of survey XPS spectra of GO and rGO.

Sample	C (at.%)	O (at.%)	N (at.%)
Graphite oxide (GO)	63.1	36.9	<0.1
Reduced graphene oxide (rGO)	94.9	3.4	1.7

In order to quantify the C/O ratio, an XPS survey was performed (Figure S6). Further we also confirmed the presence of S for GO and N for rGO. Quantification of C*1s* and O*1s* signals clearly indicates that the C/O ratio for GO is 1.7 which is in the range that is typical of GO. Further, C/O ratio for rGO significantly increases to 27.9 which is again typical for reduced graphene oxide materials. The XPS survey of rGO also revealed the presence of nitrogen that could be introduced during the reduction with hydrazine and was reported previously.

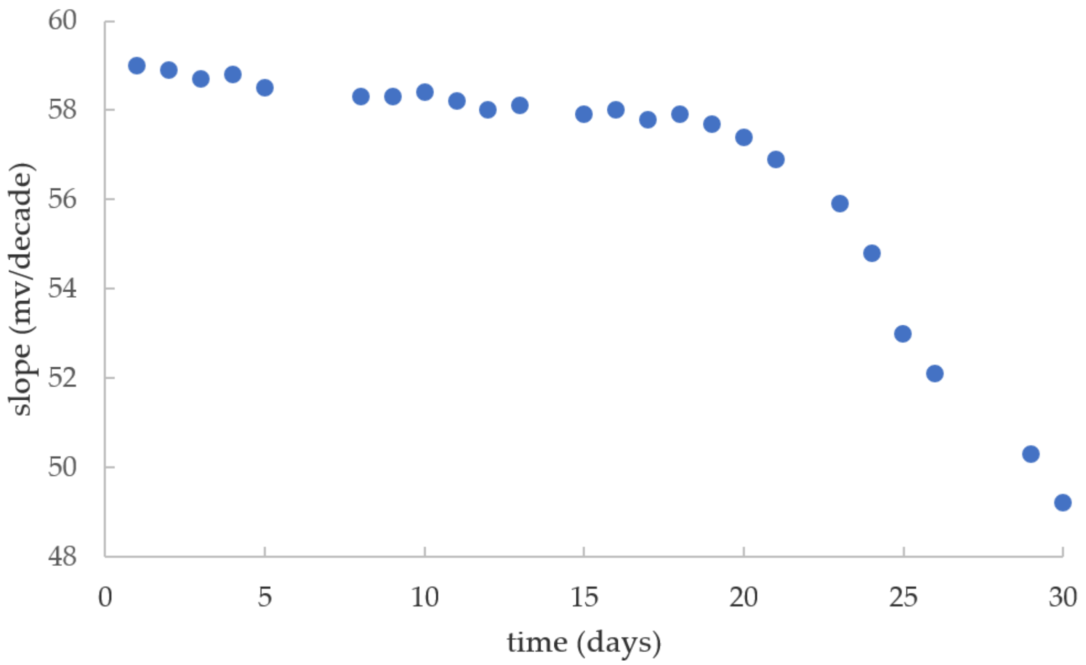

Figure 7. Change in slope value versus time.

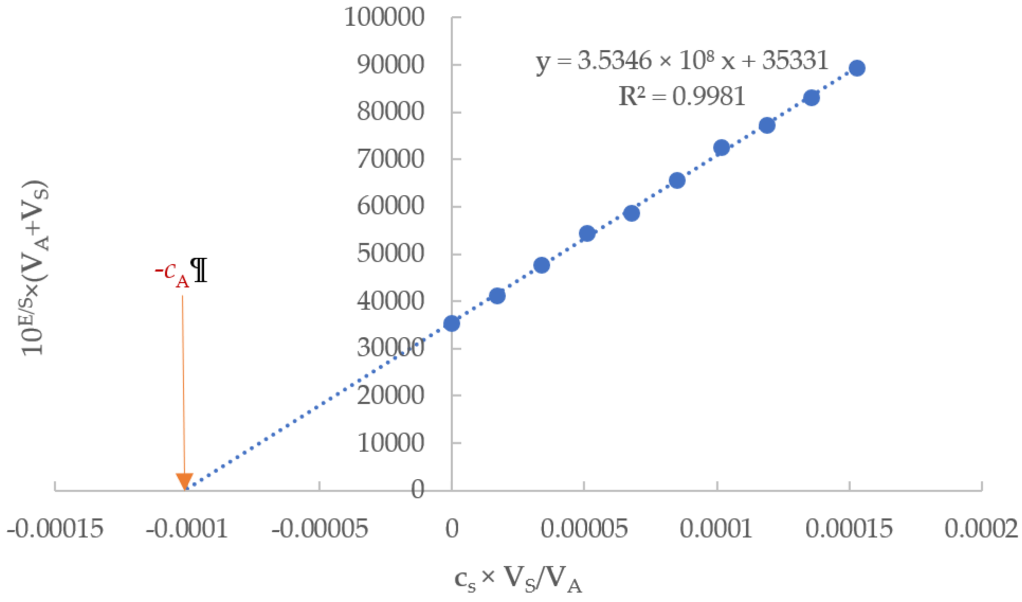

Figure 8. Standard addition method; 50 mL of urine sample aliquot using 1×10^{-3} M solution of THP as spike—standard adding solution.

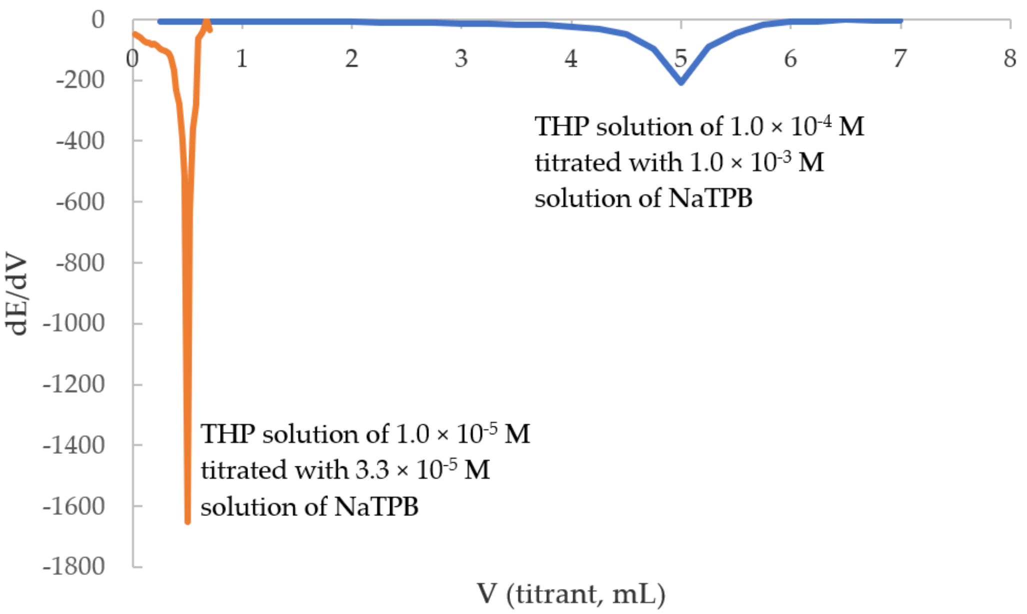

Figure 9. First order derivate curves for 50 mL aliquot of Parkopan tablets.
